# Mechanical properties and corrosion behaviour of duplex stainless steel weldment using novel electrodes

**DOI:** 10.1038/s41598-022-26974-6

**Published:** 2022-12-27

**Authors:** Ibrahim Momoh-Bello Omiogbemi, Danjuma Saleh Yawas, Atanu Das, Matthew Olatunde Afolayan, Emmanuel Toi Dauda, Roshan Kumar, Sudhakar Rao Gorja, Sandip Ghosh Chowdhury

**Affiliations:** 1 Mechanical Engineering Department, Air Force Institute of Technology, Kaduna, Nigeria; 2 Shell Professorial Chair, Mechanical Engineering Department, Ahmadu Bello University, Zaria, Nigeria; 3 Materials Engineering Division, CSIR-National Metallurgical Laboratory, Jamshedpur, India; 4 Mechanical Engineering Department, Ahmadu Bello University, Zaria, Nigeria; 5 Metallurgical and Materials Engineering Department, Ahmadu Bello University, Zaria, Nigeria; 6 Engineering Division, CSIR-National Metallurgical Laboratory, Jamshedpur, India

**Keywords:** Engineering, Materials science

## Abstract

Mechanical and corrosion properties of welded duplex stainless steel (DSS) structures are of paramount consideration in many engineering applications. The current research investigates the mechanical properties and corrosion integrity of duplex stainless-steel weldment in a simulated 3.5% NaCl environment using specially developed novel electrodes without the addition of alloying elements to the flux samples. Two different types of fluxes having basicity indexes of 2.40 and 0.40 were used to coat E1 and E2 electrodes respectively for DSS plate welding. The thermal stability of the formulated flux was evaluated using thermogravimetric analysis. The chemical composition, using optical emission spectroscopy, and the mechanical and corrosion properties of the welded joints were evaluated as per different ASTM standards. X-ray diffraction was used to find out the phases present in the DSS welded joints while a scanning electron equipped with EDS was used for microstructural examination of the weldments. The ultimate tensile strength of welded joints made using the E1 electrode was in the range of 715–732 MPa and that of the E2 electrode was found to be 606–687 MPa. The hardness was increased with increased welding current from 90 to 110 A. The welded joint with E1 electrode coated with basic flux has better mechanical properties. The steel structure in 3.5% NaCl environment possesses substantial resistance to corrosion attack. This validates the performance of the welded joints made by the newly developed electrode. The results are discussed on the basis of the depletion of alloying elements such as Cr and Mo observed from the weldments with the coated electrodes E1 and E2 as well as precipitation of the Cr_2_N in the welded joints made by E1 and E2 electrodes.

Historically speaking, the first formal reference to duplex stainless steels (DSSs) was made in 1927 and limited to some castings and was not utilized in most engineering applications due to the high carbon^1^. but the carbon content was subsequently reduced to up to 0.03% as standard and these steels are progressively being widely used for several applications^2,3^. DSSs are an alloy family that contains roughly equal amounts of ferrite and austenite. The ferrite phase in DSSs was revealed to give exceptional protection against chloride-induced stress corrosion cracking (SCC), which is a significant concern for austenitic stainless steels (ASSs), during the twentieth century. DSS demand, on the other hand, is increasing at a pace of up to 20% per year in several engineering and other industries^4^. This innovative steel, which exhibits a dual-phase constitution of austenite–ferrite, can be achieved by selecting appropriate compositions, physico-chemical and thermo-mechanical refining. In comparison to the one-phase grade of stainless steel, DSSs have higher yield strength and an exceptional ability to withstand SCC^5–8^. In harsh environments containing acids, acid chlorides, seawater, and caustic chemicals, the dual-phase structure confers unrivalled strength, toughness, and enhanced corrosion resistance to these steels^9^. DSS structures especially the low nickel-containing type (lean DSS) have recorded numerous exceptional achievements in comparison to face-centred cubic (FCC) iron due to the yearly fluctuation in nickel (Ni) alloy prices in the general market^10,11^. The main issue with ASS structures is that they are vulnerable to a variety of harsh conditions^12^. As a result, various engineering sectors and businesses are attempting to promote replacement stainless steels with reduced nickel (Ni) content that perform as well as or better than traditional ASSs having suitable weldability characteristics with application in industrial fields such as the manufacture of seawater heat exchangers and chemical containers for use in chloride environments with high concentrations^13^.

In modern technological advancement, fabrication by welding plays a critical role. Generally, the DSS steel structural components are joined by gas metal arc welding or shielded metal arc welding technique. The weld is majorly influenced by the composition of the welding electrode used for the welding. The welding electrode consists of two components; metal and flux. Most commonly the electrode is coated with flux, a metal mixture that emits gases and generates protective slag as it decomposes to shield the weld from contamination, increase arc stability, and also provide alloying components to improve weld quality^14^. Cast iron, aluminium, stainless steel, mild steel, high-tensile steel, copper, brass, and bronze are some of the weld electrode metals and cellulose, iron powder, and hydrogen are some of the flux materials that are being used. Sometimes sodium, titanium, and potassium are also added to the flux mixtures.

Some researchers have attempted to study the effect of electrode constitutions on the mechanical and corrosion integrity of welded steel structures. The influence of flux composition on the percentage elongation and tensile strength of welds in submerged arc welding was studied by Singh et al.^15^. The results demonstrate that CaF_2_ and NiO are major determinants of tensile strength as against the presence of FeMn. Chirag et al.^16^ investigated SMAW joints by varying concentrations of rutile (TiO_2_) in an electrode flux mixture. It was found that the microhardness properties increased due to the increase in the percentage and migration of carbon and silicon. Kumar^17^, studied the development and design of agglomerated fluxes in submerged arc welding for steel plate welding. The production of an arc welding flux with a potash-enriched sodium silicate-based binder was researched by Nwigbo and Atuanya^18^ and found weld having tensile strength of up to 430 MPa, with an acceptable grain structure. The corrosion behaviour of 28Cr–7Ni–O–0.34N duplex stainless steels having austenite volume fractions ranging from 0.35 to 0.64, in air-saturated 3.5-wt.% NaCl solution was examined by Lothongkum et al.^19^ using the potentiodynamic method at pH 2, 7, 10, and 27 °C. Both duplex and micro-duplex stainless steel showed a similar influence of nitrogen on corrosion behaviour. Nitrogen had no influence on corrosion potential or the rate at pH 7 and 10, however, pH 10 had a lower corrosion potential than pH 7. On the other hand, at all pH levels examined, the pitting potential of the steels rose as the nitrogen content in them increased. Lacerda et al.^20^ used a cyclic potentiodynamic polarization technique to investigate the pitting corrosion behaviour of UNS S31803 and UNS S32304 duplex stainless steels in 3.5 wt% NaCl solution. In a 3.5 wt% NaCl solution, the two plates of steel investigated showed signs of pitting corrosion. In comparison to UNS S32304 steel, UNS S31803 steel had a higher corrosion potential (E_corr_), pitting potential (E_pit_), and polarization resistance (Rp). UNS S31803 steel had a higher ability for repassivation than UNS S32304 steel. According to Jiang et al.^21^, reactivation peaks corresponding to the dual phases (austenite and ferritic phase) of duplex stainless steel include up to 65% ferrite composition, with the ferrite reactivation current density increasing with heat treatment time. At differing electrochemical potentials, the austenite and ferritic phases exhibit distinct electrochemical responses, as is well known^21–24^. Abdo et al.^25^ used polarization by potentiodynamic measurement and electrochemical impedance spectroscopy to investigate corrosion caused by electrochemistry of laser-welded 2205 DSS in an artificial seawater environment (3.5% NaCl) under different levels of acidity and alkalinity conditions. Pitting corrosion was observed on the exposed surfaces of the tested DSS samples. Based on the findings, a proportional relationship has been discovered between the pH value of the solution medium and the formed film resistance due to charge transfer processes, which has a direct impact on the pitting formation and its specifications. The goal of this study was to see how newly developed welding electrode compositions affected the mechanical and attrition integrity of welded DSS 2205 in a 3.5% NaCl environment.

## Materials and methods

### Materials

The flux minerals (ingredients) used for the formulation of the electrode coatings are calcium carbonate (CaCO_3_) was obtained from Obajana area of Kogi State-Nigeria, Calcium fluoride (CaF_2_) was obtained from Taraba state-Nigeria, silica (SiO_2_), talc (Mg_3_Si_4_O_10_(OH)_2_) and rutile (TiO_2_,) were obtained from Jos-Nigeria while kaolin (Al_2_(OH)_4_Si_2_O_5_) was obtained from Kankara in Katsina state-Nigeria. The potassium silicate was used as a binder and it was obtained from India.

### Flux formulation and design

Constituting oxides were weighed independently on a digital weighing scale as shown in Table 1. It was then mixed conscientiously in an electrically powered mixing machine (Model No: 641-048) in India Steel and Wire Product Limited (ISWP) with potassium silicate binder (23% weight) for 30 min to achieve a congruent semi-solid paste. The wet mixed flux was pressed into a cylindrical form from the briquetting machine and delivered into an extrusion chamber under a range of pressure between 80 and 100 kg/cm^2^ while a 3.15 mm diameter wire rod of the stainless steel was fed from the wire feeder chamber of the extrusion press and coated with the flux by way of nozzle/die box system incorporated into the extrusion press for electrode extrusion. A coating factor of 1.70 mm was obtained; where the coating factor is defined as the ratio of the diameter of the electrode to the core wire diameter. The coated electrodes were thereafter air-dried for 24 h before being oven-baked for 2 h at 150–250 °C $-$ in a muffle furnace (Model PH-248-0571/5448). The basicity of the flux was calculated using Eq. (1)^26^;

1 $${\text{B}}.{\text{I}}. \, = \frac{{{\text{CaO}} + {\text{MgO}} + {\text{K}}_{2} 0 + {\text{CaF}}_{2} + 0.5\left( {{\text{MnO}} + {\text{FeO}}} \right)}}{{{\text{SiO}}_{2} + 0.5\left( {{\text{Al}}_{2} 0_{3} + {\text{Ti}}0_{2} } \right){ }}}$$

**Table 1:** Flux design for extrusion.

Flux ingredients	CaF_2_ (wt.%)	CaCO_3_ (wt.%)	SiO_2_ (wt.%)	TiO_2_ (wt.%)	Talc (wt.%)	Kaolin (wt.%)	Basicity index (B.I.)
F1	25.00	35.00	10.00	25.00	1.00	4.00	2.40
F2	5.00	15.00	20.00	50.00	2.00	8.00	0.40

### Thermogravimetric analysis (TGA) of formulated fluxes

The thermal stability of the formulated flux samples for E1 and E2 was determined using thermogravimetric analysis (TGA). Approximately 25.33 mg of flux sample was loaded into the TGA equipment for analysis. The experiment was carried out in an inert environment obtained by the continuous flow of 60 mL/min of N_2_. Samples were heated from 30 to 1000 °C at heating rates of 10 °C/min. The thermal decomposition, as well as samples’ weight loss at specific temperatures, were evaluated from the TGA graphs following the approaches mentioned by Wang et al.^27^, Xu et al.^28^, and Dagwa et al.^29^.

### Experimental weld design and procedure

Two plates of DSS having 300 × 60 × 6 mm dimensions were machined in preparation for welding. A V-groove weld design with a root gap of 3 mm, root opening of 2 mm, and a groove angle of 60° was made. The plate was thereafter cleaned with acetone to get rid of any possible dirt. Shielded metal arc welding (SMAW) machine with direct current electrode positive (DCEP) polarity was used to weld the plates using 3.15 mm diameter of the coated electrodes (E1 and E2), and control electrode (C). Electric discharge machining (EDM) (Model: Excetek-V400) was used to machine the samples for mechanical testing and corrosion characterization from the welded steel. Table 2 presented sample codes and their description while Table 3 shows the different operational welding parameters used to weld DSS plates. Equation (2) was used to calculate the heat input accordingly^30^.

2 $${\text{Heat input }}\left( {{\text{kJ}}/{\text{mm}}} \right) = \frac{{{\text{Voltage }} \times {\text{Amperage }} \times 60}}{{1000 \times {\text{Travel speed }}\left[ {{\text{mm}}/{\text{min}}} \right]}}$$

**Table 2:** Experimental samples labelling.

Sample no	Sample code	Remark
1	DSS BM	Duplex stainless steel base metal (AISI 2205)
2	DSS E1 @90A	DSS plate welded with E1 electrode at 90A
3	DSS E1 @110A	DSS plate welded with E1 electrode at 110A
4	DSS E2 @90A	DSS plate welded with E2 electrode at 90A
5	DSS E2 @110A	DSS plate welded with E2 electrode at 110A
6	C DSS @90A	DSS plate welded with control electrode at 90A
7	C DSS @110A	DSS plate welded with control electrode at 110A

*F1* Flux ingredients for basic electrode, *F2* Flux ingredients for acidic electrode.

**Table 3:** Operating welding conditions for E1, E2, and C welded joints.

DSS plate	Electrodes used	Current (A)	Voltage (V)	Speed (mm/min)	Heat input (kJ/mm)
2205	C	90	22.5–28.0	107.78	1.13
		110	22.5–28.0	98.36	1.51
2205	E1	90	22.5–28.0	97.83	1.24
		110	22.5–28.0	112.36	1.32
2205	E2	90	22.5–28.0	80.00	1.52
		110	22.5–28.0	109.00	1.36

### Spectrophotometry analysis

Using the Bruker Q8 MAGELLAN optical emission spectroscopy (OES) with a wavelength between 110 and 800 nm and SQL database software, the chemical composition of the welded joints with E1, E2, and C electrodes, as well as the base metal sample, was determined. OES uses electrical energy in the form of a spark generated between an electrode and the research metal sample. The constituents’ sample is vaporized and atomized, followed by the atoms excitation that subsequently emits particular line spectra^31^. To perform the qualitative analysis of the samples, the photomultiplier tubes measure the presence of the extracted spectrum for each element as well as the intensity of the spectrum. Thereafter, the pitting resistance equivalent number (PREN) was calculated using Eq. (3) relation^32^ while the WRC 1992 Constitution Diagram was used to calculate the chromium and nickel equivalents (Cr_eq_ and Ni_eq_) from Eqs. (4), and (5) respectively^33,34^;

3 $${\text{PREN}} = \% {\text{Cr}} + {3}.{3}*\left( {\% {\text{Mo}}} \right) + {\text{x}}*\left( {\% {\text{N}}} \right)$$

4 $${\text{Cr}}_{{{\text{eq}}}} = \% {\text{Cr}} + \% {\text{Mo}} + 0.{7}*\left( {\% {\text{Nb}}} \right)$$

5 $${\text{Ni}}_{{{\text{eq}}}} = \% {\text{Ni}} + {35}*\left( {\% {\text{C}}} \right) + {2}0*\left( {\% {\text{N}}} \right) + 0.{25}*\left( {\% {\text{Cu}}} \right)$$

Note that PREN only takes into account the positive effects of the major three elements, Cr, Mo, and N, while the nitrogen factor x is in the range of 16–30. Typically, x is picked from a list of 16, 20, or 30. In the study for duplex stainless steels, a middle value of 20 is most frequently used to calculate the PREN value^35,36^.

### Mechanical properties

#### Tensile test

Tensile test of the welded joints produced using the various electrodes was carried out according to ASTM E8-21 standard by a universal testing machine (Instron 8800 UTM), at a strain rate of 0.5 mm/min. The ultimate tensile strength (UTS), 0.2% offset yield strength (YS) and percentage elongation was calculated according to ASTM E8-21 standard^37^.

#### Micro-hardness test

The welded DSS 2205 sample was initially ground and polished using different size grits (120, 220, 320, 400, 600, 800, 1000, and 1200) before hardness analysis. Vickers hardness tester (Omnitech make, model F.-AUTO) with a test load of 1 kgf was employed to carry out the hardness test as per ASTM E92-17 standard^38^ (˂ 1- ≤ 120 kg) on the welded samples made using E1, E2, and C electrodes. The hardness was measured at ten (10) locations from the centre of the weld towards the base metal with an interval of 1 mm.

### Phase analyses and microstructural characterization

The X-ray diffractometer (D8 Discover, Bruker, Germany) configured with Bruker XRD commander software for data acquisition and Fe filtered Cu–K-α radiation having an energy of 8.04 keV with a corresponding wavelength of 1.5406 Å and a scan speed of 3° min^-1^ having a scan range (2θ) between 38 and 103° was used to analyze the phases present in the DSS welded joints with E1, E2 and C electrodes along with BM. The Rietveld refinement method was used to index the constituent phases using the MAUD software described by Lutterotti^39^. Based on ASTM E1245-03, quantitative metallographic analysis of the microscopy images of welded joints with E1, E2, and C electrodes was carried out using Image J software^40^. The volume fractions of the ferrite–austenite phases with their averages and deviations were calculated as shown in Table 5 in the results. The optical microscope (OM) analysis was carried out on the BM and welded joints with E1 and E2 electrodes for morphological studies of the samples as indicated in the sample configuration in Fig. 6d. The samples were polished with silicon carbide (SiC) paper grits of 120, 220, 320, 400, 600, 800, 1000, 1200, 1500, and 2000. Thereafter, they were electrolytically etched in 10% aqueous oxalic acid at room temperature at a voltage of 5 V for 10 s and mounted on LEICA DM 2500 M optical microscope for morphological characterisations. A further sample polish with silicon carbide (SiC) paper grits of 2500 for SEM-BSE analysis was done. In addition, an ultra-high-resolution field-emission scanning electron microscope (SEM) machine (FEI NOVA NANOSEM 430, USA) equipped with EDS was used to carry out the microstructural examination of the weldments. Sample configuration of 20 × 10 × 6 mm was grounded using different SiC sand-paper grit of sizes from 120 to 2500. The sample was etched in 40 g of NaOH with 100 ml of distilled water electrolytically at a voltage of 5 V for 15 s and thereafter mounted on the sample holder located in the SEM chamber for sample analysis after the chamber was purged with N_2_. The electron beam generated from a heated tungsten filament was rastered over the sample to produce images of different magnifications with EDS results obtained adopting the approaches of Rocha et al.^41^ and Mokobi^42^.

### Corrosion assessment

The electrochemical technique as per ASTM G59–97 standards^43^ and ASTM G5–14 standards^44^ for potentiodynamic polarization was used to evaluate the deterioration potentials of the welded DSS 2205 plate with E1, E2, and C electrodes in a 3.5% NaCl environment. The electrochemical tests were carried out using a computer-controlled Potentiostat–Galvanostat/ZRA apparatus (Model: PC4/750, Gamry Instruments, USA). Electrochemical tests were performed with three electrode test setups: the DSS 2205 as the working electrode, a saturated calomel electrode (SCE) as the reference electrode, and a graphite rod as the counter electrode. An electrochemical cell was used for the measurements in which the area of the exposed region to the solution is 0.78 cm^2^ of the working electrode. At a scan rate of 1.0 mV/s, the measurements were made between the potentials of − 1.0 V and + 1.6 V on a pre-stabilized OCP (w.r.t OCP).

#### Critical pitting temperature tests (CPT)

The electrochemical critical pitting temperature tests were carried out in 3.5% NaCl to evaluate the pitting corrosion resistance of the welds prepared with E1, E2, and C electrodes. It is important to mention here that, the potentiodynamic polarization test results (discussed in a later section in Fig. 11b) revealed no clear indication of pitting potentials (between passive and trans-passive regions) in the BM, and welded samples with E1, E2, and C electrodes. Hence, CPT measurements were carried out to exactly determine the pitting potentials of the weld materials. The CPT experiments were done in accordance with the reported literature on duplex stainless steel welds^45^ and ASTM standard G150–18^46^. Specimens with surface area of 1 cm^2^ consisting of base, weld and HAZ regions were sectioned from each welded steel (C-110A, E1-110A and E2-90A). The samples were polished following the standard metallographic specimen preparation procedures using emery papers and 1 µm alumina powder suspension. After polishing the specimens were ultrasonic cleaned in acetone for 2 min. The 3.5% NaCl test solution is added in the CPT test cell and the initial temperature was adjusted to 25 °C  using thermostat (Neslab RTE-111). After reaching to the initial test temperature of 25 °C Ar gas was purged for 15 min, then sample was placed in the cell and OCP was measured for 15 min. Subsequently at the initial temperature of 25 °C the sample was polarized by applying the 0.3 V and current was measured for 10 minutes^45^. The heating of the solution was started at 1 °C/minute up to 50 °C. During heating of the test solution, the solution temperature was continuously monitored using a temperature sensor and stored the time verses temperature data, at the same time current was measured using a potentiostat/galvanostat. A graphite electrode has been used as counter electrode and all potentials were measured relative to a Ag/AgCl reference electrode. Argon gas purging was done throughout the test.

## Results and discussion

### Flux characterization

**Figure 1:**
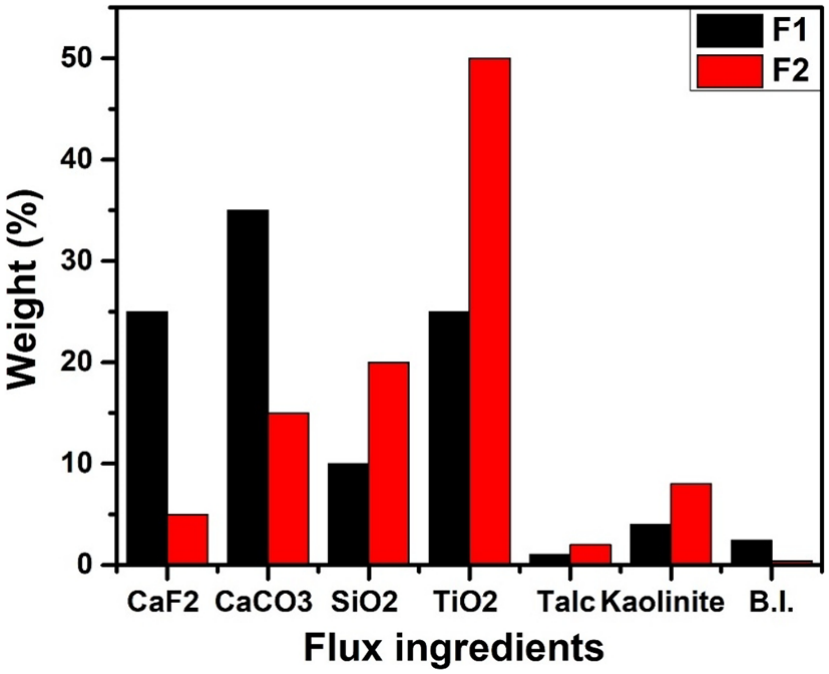
Flux ingredients for SMAW electrode coating.

Figure 1 shows the composition of F1 and F2 flux ingredients (wt.%) used in the production of basic (E1) and acidic (E2) electrodes respectively. The flux basicity index is used to predict the mechanical and metallurgical properties of the welded joint. F1 is the flux ingredient used for E1 electrode coating and is termed basic flux because it has a basicity index > 1.2 (i.e., 2.40) while the F2 is a flux used for E2 electrode coating is called acidic flux because it has a basicity index < 0.9 (i.e., 0.40). It is evident that electrodes coated with basic flux possess good mechanical properties than electrodes coated with acidic flux in most scenarios. This property is a function of the basic oxides’ dominance in the flux formulation system of the E1 electrode. Conversely, slag removability (detachability) and low weld spatter observed with welded joints with E2 electrodes is a characteristic of acidic flux-coated electrodes with high rutile contents. This observation corresponds with the findings of Gill^47^ on the influence of rutile contents on slag detachability and low weld spatter in coated electrodes with acidic flux which helps to facilitate the quick freezing of the slag. Kaolin in the flux system used in coating electrodes E1 and E2 serves as a slippery agent and talc improves electrode extrudability. Potassium silicate binder in the flux system helps to achieve better arc striking and stability characteristics and also enhances slag detachability in the weldment in addition to its binding-ability property. Since CaCO_3_ is a network breaker (slag breaker) in flux formulation and tends to produce a lot of fumes during welding by thermally decomposing into CaO and approximately 44% CO_2_, TiO_2_ addition (as a network former/slag former) in the flux constituent helps to reduce fumes during welding thereby enhancing slag detachability as opined by Jing et al.^48^. The fluoride content in the flux (CaF_2_) is a chemically aggressive fluxing agent that improves weld cleanliness. This kind of flux ingredients composition was reported by Jastrzębska et al.^49^ on the influence of fluoride composition on weld cleanliness characteristics. Generally, the addition of fluxes to the welding domain is to improve arc stability, add alloying elements, provide slag, increase productivity and refine the weld pool^50^.

### TGA characterization of formulated fluxes

**Figure 2:**
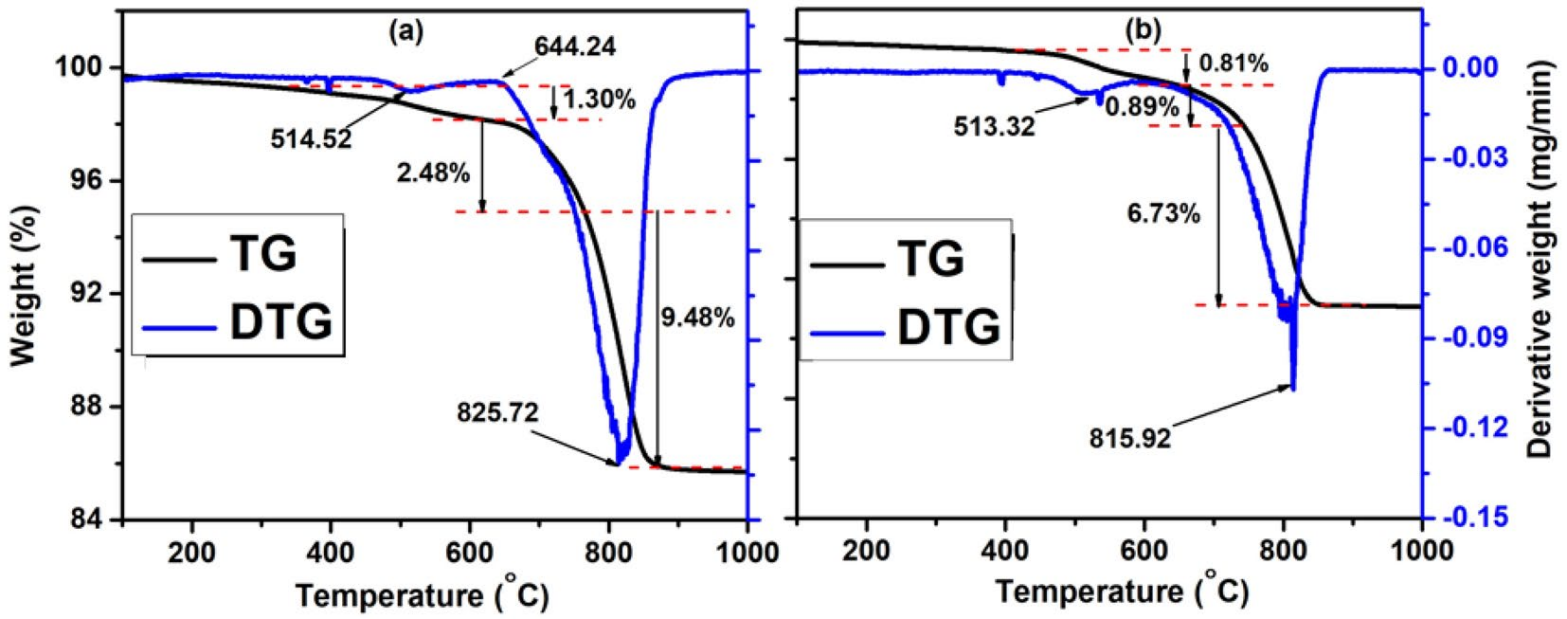
TGA-DTG curves showing thermal decompositions of (**a**) basic flux for E1 electrode coating and (**b**) acidic flux for E2 electrode coating.

The TGA-DTG curves presented in Fig. 2a and b reveal a three-step weight loss on heating in the temperature range of 30–1000 °C under a nitrogen gas environment. The results in both Fig. 2a and b show that the TGA curve plummets until it eventually becomes parallel to the temperature axis at around 866.49 °C and 849.10 °C respectively for basic and acidic flux samples. The 1.30 and 0.81% weight loss experienced at the start of the TGA curve in Fig. 2a and b were due to evaporation and dehydration of the absorbed and surface moisture from the flux ingredients. The second and third stages of major decompositions of the basic flux sample in Fig. 2a happen in the temperature ranges of 619.45 °C–766.36 °C and 766.36 °C–866.49 °C with their percentage mass loss of 2.84 and 9.48% respectively. While for the acidic flux sample in Fig. 7b occurs in the temperature ranges of 665.23 °C–745.37 °C and 745.37 °C–849.10 °C with their percentage mass loss of 0.81 and 6.73% respectively which is attributed to the thermal decomposition of volatile matters capped in the flux mixtures since the flux ingredients are inorganic matters. Hence, reduction and oxidation were appalling. This is in consonant with the results of Balogun et al.^51^, Kamli et al.^52^ and Adeleke et al.^53^. The summation of the observed mass loss in the flux samples in Fig. 2a and b are respectively 13.26 and 8.43%. The less mass loss noticed with the flux sample in Fig. 2b is due to the high melting point of TiO_2_ and SiO_2_ (1843 and 1710 °C respectively) as the major constituent oxides in the flux mixture^54,55^ as against the low melting temperature of the dominant oxide: CaCO_3_ (825 °C) in the flux sample in Fig. 2a^56^. These variations in melting temperatures of the principal oxides in the flux mixtures have been well reported by Shi et al.^54^, Ringdalen et al.^55^, and Du et al.^56^. After a successive weight loss observed in Fig. 2a and b, it can be concluded that the flux samples used in E1 and E2 electrode coatings experienced a single-stage decomposition as posited by Brown^57^. The temperature ranges of the process can be seen from the derivative (wt.%) curve in Fig. 2a and b. Since the TGA curve cannot exactly tell the specific temperature where phase transformation and crystallinity of the flux system occur, the derivative of the TGA was taken to determine the exact temperature value in the form of the endothermic peak for each phenomenon (phase transformation) in the formulated flux system.

### Optical emission spectroscopy (OES) and SEM–EDS analyses

**Figure 3:**
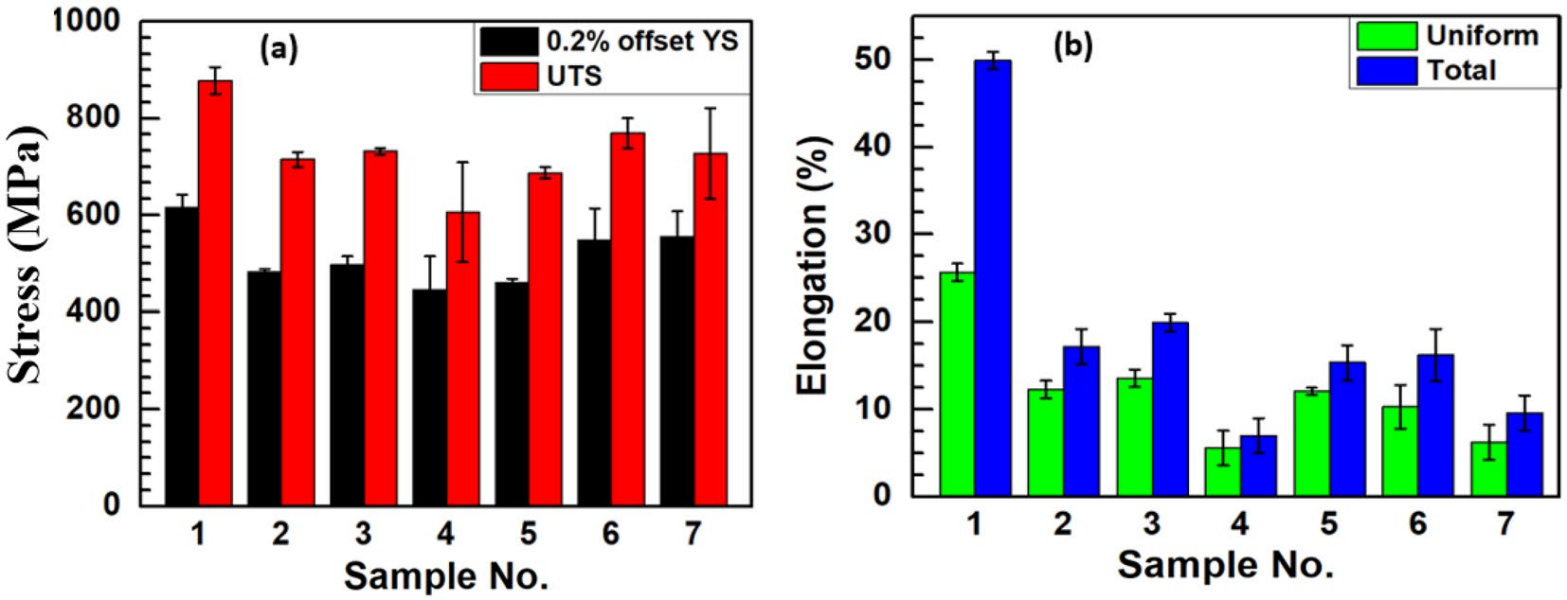
Tensile test results (**a**) UTS and 0.2% offset YS and (**b**) uniform and total elongation with their standard deviations.

Table 4 presents the spectrophotometry as well as the SEM–EDS analyses of DSS 2205 base metal and the welded joints made using E1, E2, and C electrodes. The composition of the base plate of 2205 falls under the ASTM A240 standard of duplex stainless steel as shown in Table 4. Compositional analysis of E1 and E2 show a sharp decrease in chromium (Cr) content to 18.94 and 17.04%, and Molybdenum (Mo) content to 0.06 and 0.08%, respectively which have significantly contributed to the low values of PREN of the welded joints with E1 and E2 electrodes as compared to the welded joint made by C electrode. This slightly corresponds with the calculated values of PREN of ferrite/austenite phases from the SEM–EDS analysis. Consequently, it is evident that pitting initiation begins at the phase with low PREN values (welded joints made by E1 and E2) mostly as mentioned in Table 4. This indicates depletion and possible precipitation of the alloys in the weld. Subsequently, a decrease in the Cr and Mo alloy contents in the weldment developed using E1 and E2 electrodes and their low pitting resistance equivalent number (PREN) shown in Table 4 poses a challenge to maintaining resistance in corrosive environments, especially chloride-containing environments. The relatively high nickel (Ni) contents of 11.14% and acceptable limit of manganese contents in the welded joints made by E1 and E2 electrodes could have contributed positively to the mechanical properties of the welded structures used in a simulated seawater environment (Fig. 3). This finding can be compared to the works of Yuan and Ou^58^, and Jing et al.^48^ on the effect of high Ni and Mn compositions in improving the mechanical behaviours of welded DSS structures in a harsh service environment.

**Table 4:** Chemical composition of base metal (BM) and electrodes (in wt.%), Cr_eq_/Ni_eq_ ratio (based on WRC-1992), SEM–EDS of Austenite (A) and Ferrite (F) parts of BM, welded joints with C, E1, and E2 electrodes and their PREN values accordingly.

Chemical composition of base metal (BM) and electrodes (in wt.%), Cr_eq_/Ni_eq_ ratio (based on WRC-1992)															
BM and weld metal	C	Si	Mn	Cr	Ni	Mo	P	S	Cu	N	Fe	Cr_eq_	Ni_eq_	Cr_eq_/Ni_eq_	PREN
2205 BM	0.030	0.40	0.86	22.97	4.85	2.97	0.02	0.002	0.15	0.17	Bal	25.94	9.34	2.78	36.171
Joint made by C	0.030	0.61	0.92	21.65	9.43	3.31	0.02	0.004	0.12	0.14	Bal	24.96	13.31	1.88	35.373
Joint made by E1	0.030	0.19	0.80	18.94	11.14	0.06	0.02	0.010	0.07	0.05	Bal	19.00	13.20	1.44	20.138
Joint made by E2	0.030	0.67	0.49	17.04	11.81	0.08	0.02	0.010	0.05	0.07	Bal	17.12	14.27	1.20	18.704
Wire rod for E1 & E2	0.030	0.90	1.02	23.00	7.50	3.05	0.03	0.020	0.75	0.19	Bal	26.05	12.54	2.08	36.865
Wire rod for C	0.028	0.52	0.81	23.21	6.75	2.86	0.02	0.005	0.31	0.12	Bal	26.07	10.28	2.54	35.048

SEM–EDS analysis of Austenite (A) and Ferrite (F) phases															
BM and weld metal	C	Si	Mn	Cr	Ni	Mo	P	S	Cu	N	Fe	PREN			
2205 BM (A)	1.61	0.61	0.82	23.45	3.85	3.94	0.20	0.11	0.47	0.18	Bal	40.051			
2205 BM (F)	1.43	0.62	1.13	21.76	5.53	3.17	0.26	0.00	0.83	0.16	Bal	35.421			
Joint C (A)	1.41	0.95	1.50	21.54	8.08	3.88	0.00	0.00	1.77	0.20	Bal	38.144			
Joint C (F)	1.40	0.61	1.83	20.73	5.96	3.67	0.00	0.00	1.52	0.11	Bal	35.041			
Joint E1 (A)	1.41	0.42	1.06	21.51	5.19	2.32	0.19	0.00	1.44	0.12	Bal	31.566			
Joint E1 (F)	1.36	0.54	1.23	18.77	7.21	1.07	0.50	0.00	2.01	0.10	Bal	24.301			
Joint E2 (A)	1.38	0.91	0.64	18.69	9.44	1.43	0.09	0.07	0.99	0.11	Bal	24.652			
Joint E2 (F)	1.59	0.78	0.28	17.56	7.75	0.95	0.50	0.15	1.96	0.09	Bal	20.695

### Mechanical properties

#### Tensile properties

The tensile properties of the base material (BM) and welded joints made by the developed electrodes (E1 and E2), and the commercially purchased electrode (C) were evaluated using two different welding currents of 90 A and 110 A. Figures 3 (a) and (b) present the UTS, 0.2% offset YS and their elongations with standard deviations data. The UTS and 0.2% offset YS results obtained from Fig. 3a shows optimum values for sample No.1 (BM), sample No.3 (welded joint with E1), sample No.5 (welded joint with E2), and sample No.6 (welded joint with C) are respectively 878 and 616 MPa, 732 and 497 MPa, 687 and 461 MPa, and 769 and 549 MPa with their respective standard deviations. It is evident in Fig. 3a that the BM as well as the welded joints with E1 electrode in the two welding conditions (90 A and 110 A); E2 electrode at 110 A and C electrodes in the two welding conditions (90 A and 110 A) representing Samples No. 1, 2, 3, 6, and 7 respectively, possess beyond the minimum recommended tensile properties of 450 MPa YS and 620 MPa UTS as posited by Grocki^32^. The percentage elongations of the welded samples with E1, E2, and C electrodes represented by samples No. 2, 3, 4, 5, 6, and 7 at welding currents of 90 A and 110 A respectively depict the ductility and soundness of weldment in relation to the base metal. The lower elongation is attributed to possible welding defects or due to flux compositions of the electrode (Fig. 3b). It can be concluded that duplex stainless-steel BM and welded joints with E1, E2, and C electrodes generally demonstrated significantly higher tensile characteristics due to their relatively higher Ni (Table 4) but this characteristic was observed to be less effective in the welded joints with E2 obtained from acidic flux ingredients. The assertion is corroborated by Gunn^59^ on the influence of Ni-alloy in improving the mechanical properties as well as controlling phase balance and element partitioning in welded joints. This further verifies the fact that developed electrodes from basic flux ingredients exhibit better mechanical properties than developed electrodes from acidic flux mixtures as opined by Bang et al.^60^. Hence, a significant contribution to existing knowledge on the performance of the welded joint with the newly coated electrode (E1) having good tensile properties.

#### Micro-hardness test analysis

**Figure 4:**
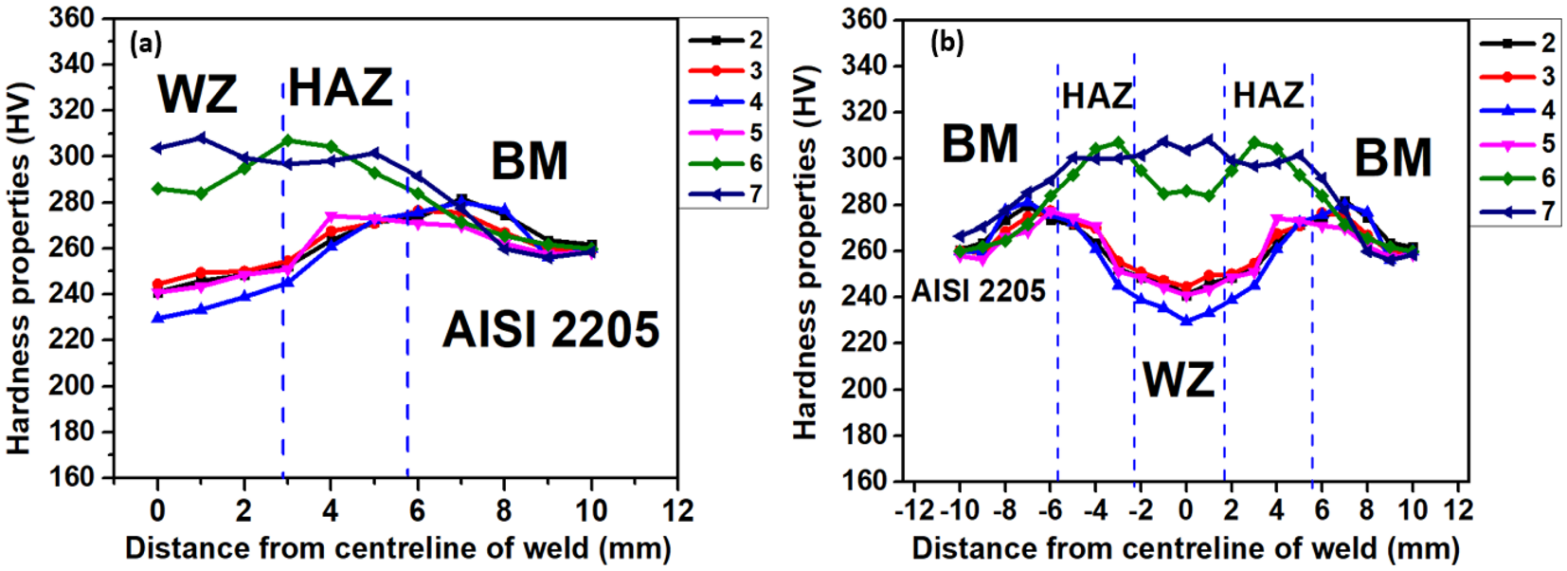
Hardness values of welded joints of DSS samples (**a**) half segment of the welded sample and (**b**) full segment of the welded joint.

Figure 4a and b show the experimented samples of Vickers micro-hardness characteristics of welded joints with E1, E2, and C electrodes. Figure 4a indicates the hardness results obtained from one direction of the sample (from WZ to BM) while Fig. 4b shows the hardness results obtained from both sides of the sample transversely. The hardness values acquired at the weldment for samples No. 2, 3, 4, and 5 representing the welded joint with E1 and E2 electrodes could be due to the coarse grain constitution in the course of the weld cycle solidification. A sharp increase in hardness was seen at the coarse grain-HAZ and fine grain-HAZ of all samples No. 2-7 (see sample code in Table 2), can be accredited to the possible change in the weld microstructure resulting in the chromium-rich precipitate (Cr_23_C_6_) of the welded samples. The hardness values of the welded joints of samples No. 6 and 7 in Fig. 4a and b are more as compared to the other welded samples No. 2, 3, 4, and 5 (Table 2). This can be attributed to the high delta ferrite and induced residual stresses in the weldment as well as the depletion of alloying elements components such as Mo and Cr in the welds as asserted by Mohammed et al.^61^, and Nowacki and Lukoje^62^. There seems to be consistency in the hardness values of all the considered samples of experimentation at the BM zone. The trend of the results of the hardness analysis of the welded samples is consistent with the findings of other investigators^61,63,64^.

### X-ray diffraction and quantitative metallography of the welded joints

**Figure 5:**
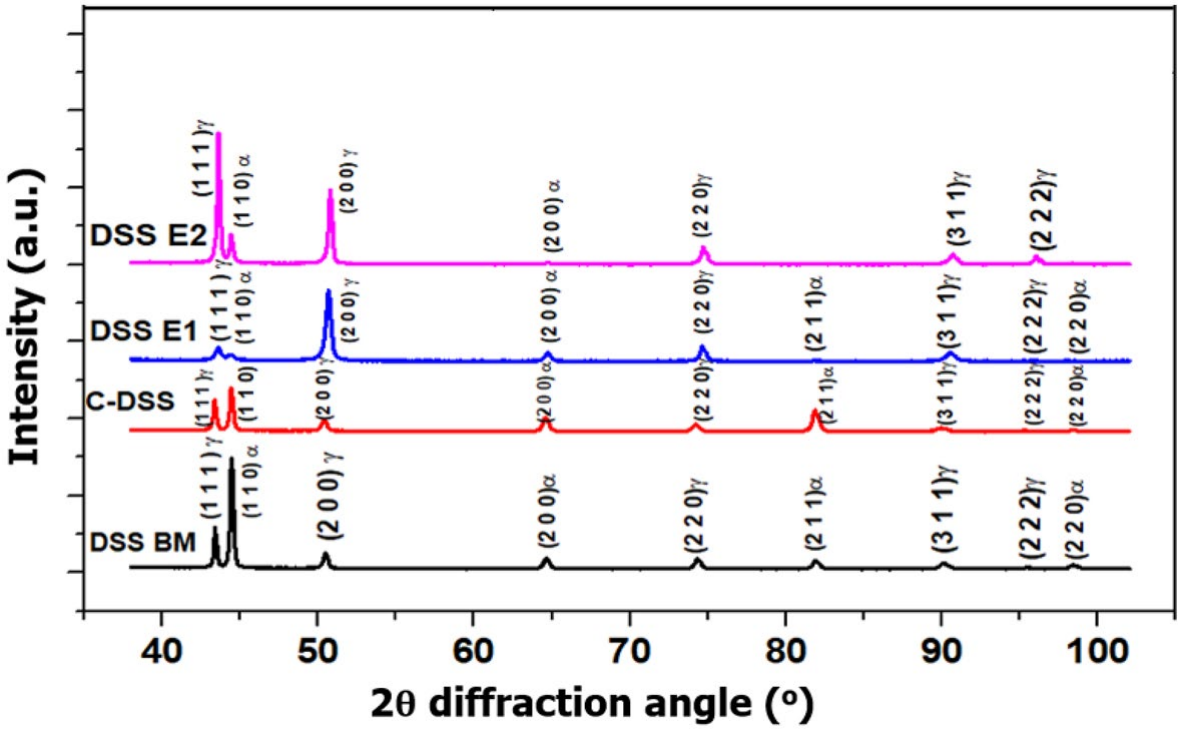
XRD spectra of welded joints of DSS 2205 with E1, E2, C electrodes, and BM.

**Figure 6:**
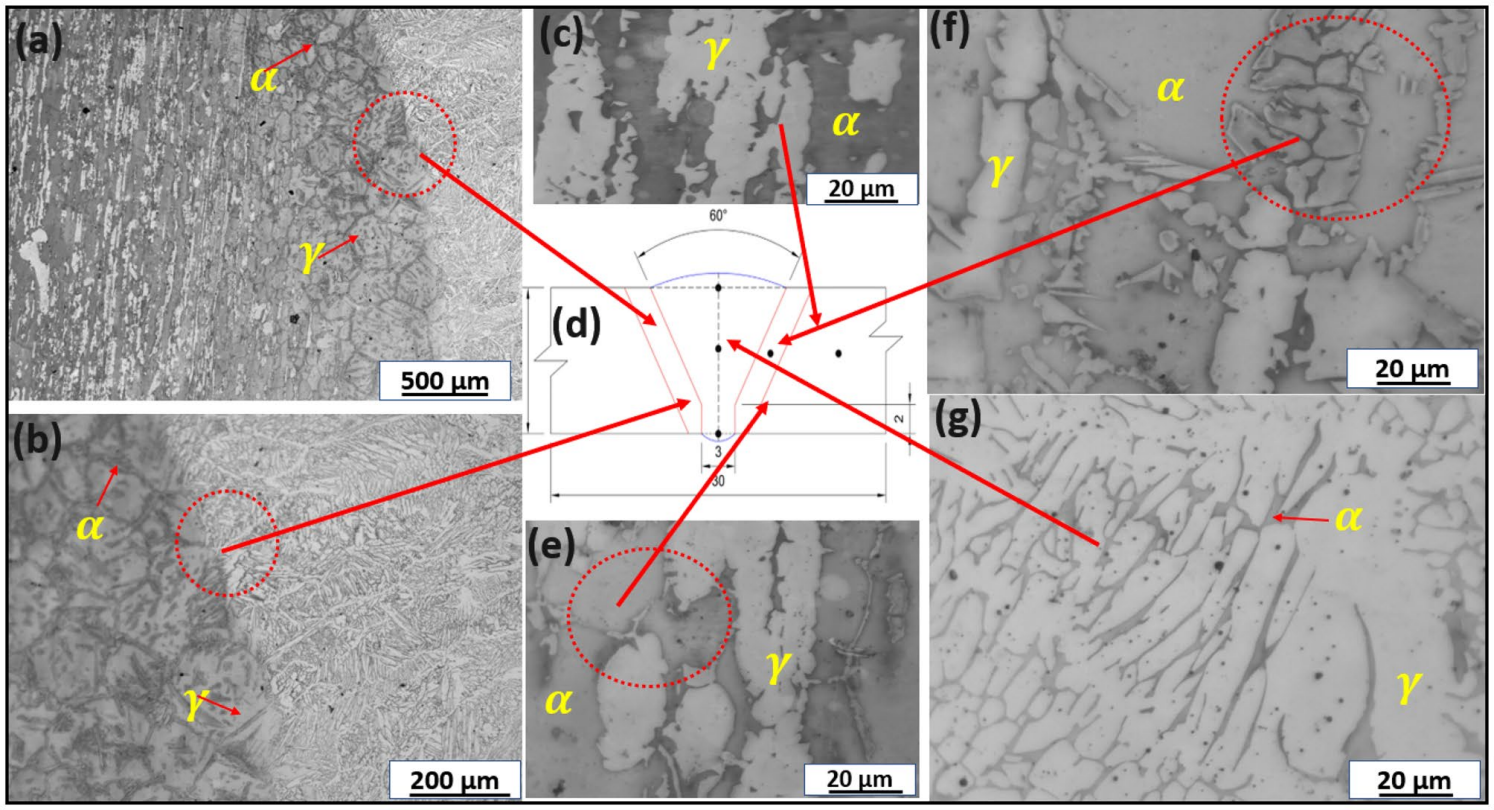
Optical micrograph of welded joint with E1 electrode at various weld geometries: (**a**) HAZ showing fusion line, (**b**) HAZ showing fusion line in higher magnification, (**c**) BM indicating ferrite–austenite phases (**d**) geometry of weld, (**e**) showing near transition zone, (**f**) HAZ showing ferrite–austenite phases in higher magnification and (**g**) weld zone showing ferrite–austenite phases.

**Figure 7:**
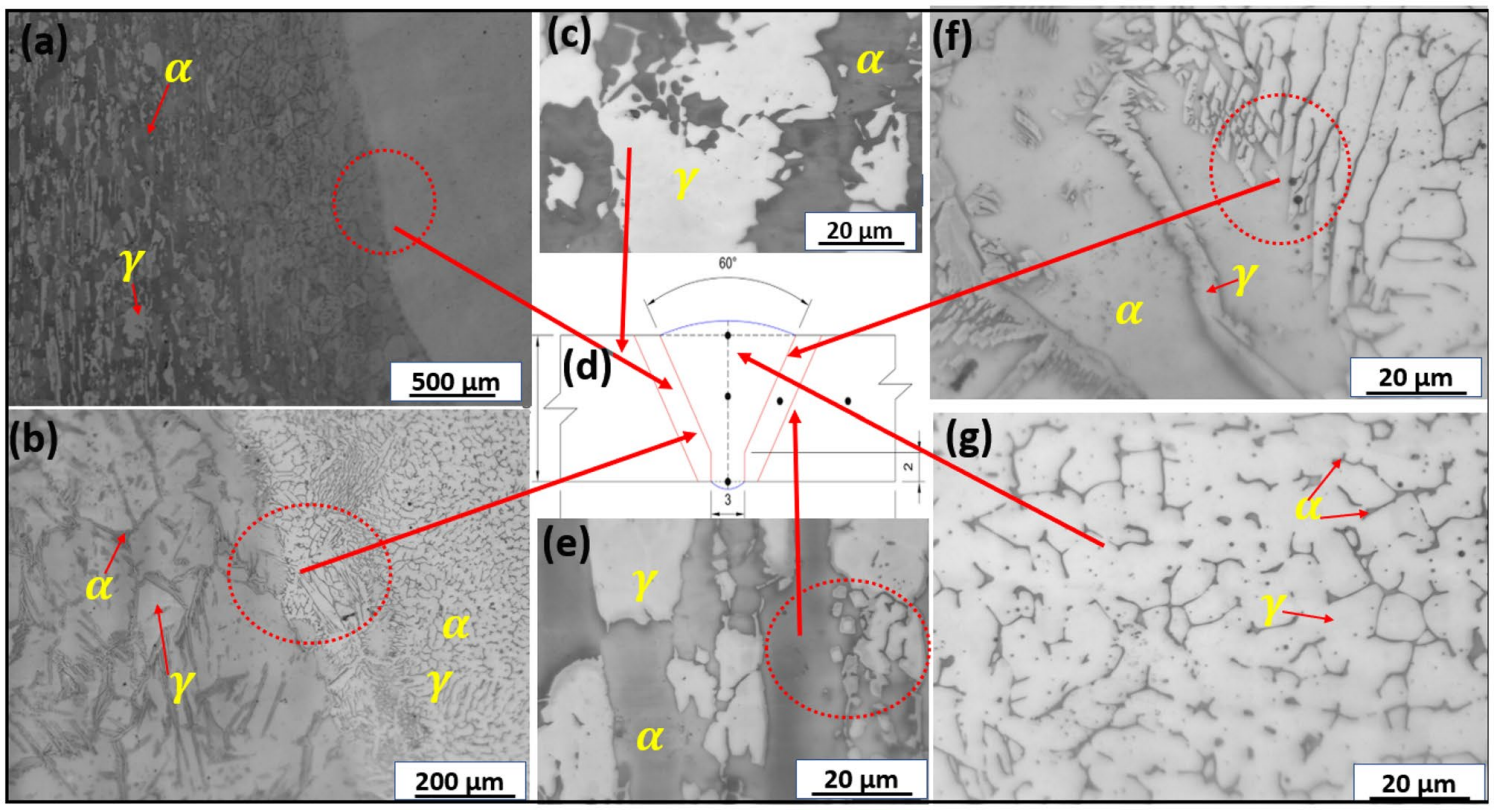
Optical micrograph of welded joint with E2 electrode at various weld geometries: (**a**) HAZ showing fusion line, (**b**) HAZ showing fusion line in higher magnification, (**c**) BM indicating ferrite–austenite phases (**d**) geometry of weld, (**e**) showing near transition zone, (**f**) HAZ showing ferrite–austenite phases in higher magnification and (**g**) weld zone showing ferrite–austenite phases.

The various phases present in the welded DSS 2205 with E1, E2, and C electrodes were obtained and the XRD spectra at 2$\theta$ diffraction angle are shown in Fig. 5. The peaks of austenite ($\gamma$) and ferrite ($\alpha$) phases identified at 43 and 44° diffraction angles, which ultimately validate the compositions of the weldment as dual-phase stainless steel as posited by Jimenez et al.^65^. It can be seen that the DSS BM only reveal the austenite ($\gamma$) and ferrite ($\alpha$) phases, authenticating the microstructural results given in Figs. 6c, 7c, and 9c. The high peaks of the ferrite ($\alpha$) phase observed with the DSS BM and in the welded joint with the C electrode are evidence of its corrosion resistance capability as this phase is meant to promote the steel’s corrosion resistance due to the presence of ferrite stabilizing elements such as Cr and Mo which effectively stabilizes the passive film of the material in a chloride-containing environment as asserted by Davison and Redmond^66^. Table 5 shows the ferrite–austenite phases using quantitative metallography. The ratio of volume fractions of ferrite–austenite phases of welded joint with C electrode approximately attains (≈ 1:1). The low ferrite ($\alpha$) phase compositions seen in weldment with E1 and E2 electrodes in the volume fraction results (Table 5) show its possible susceptibility to a corrosive environment as corroborated by the electrochemical analysis (Fig. 10a, b) because the ferrite phase offers high strength and protection against stress corrosion cracking caused by chloride. It was further verified by the low hardness values observed in the weldments with E1 and E2 electrodes from Fig. 4a, b caused by their low ferrite fractions in the steel structure (Table 5). The presence of unbalanced austenite ($\gamma$) phase and ferrite ($\alpha$) phase in weldment with an E2 electrode is an indication of the practicable vulnerability of the steel to uniform corrosion attack. Conversely, the XRD spectra of the dual phases steel of the weldments with E1 and C electrodes as well as the BM results, generally show the presence of austenite and ferrite stabilizing elements that made the material find applications in both structural engineering and petrochemical industries as asserted by Jimenez et al.^65^; Davison and Redmond^66^; Shamanth et al.^67^.

**Table 5:** Volume fractions of research samples from quantitative metallography analysis.

Samples	Volume fraction from quantitative metallography (%)	
	Austenite (FCC)	Ferrite (BCC)
Weld joint made by C	49.351 ± 2.99	50.646 ± 0.75
Weld joint made by E1	71.371 ± 4.41	28.629 ± 1.05
Weld joint made by E2	72.052 ± 7.93	27.948 ± 1.20

### Optical microscope and SEM characterization of the welded joints

**Figure 8:**
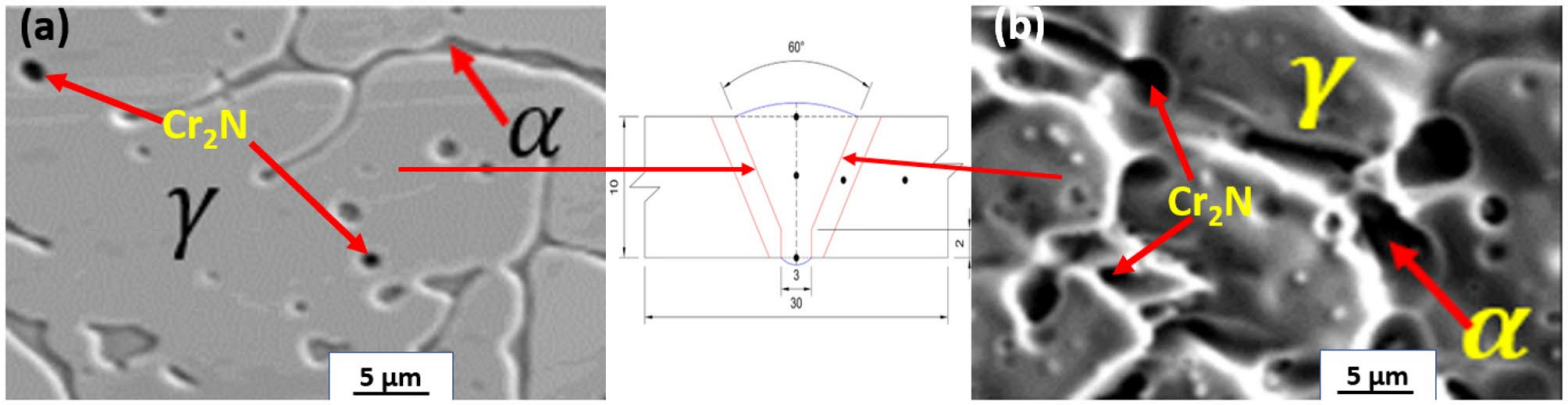
SEM-BSE micrographs of (**a**) welded joint with E1 electrode and (**b**) welded joint with E2 electrode.

**Figure 9:**
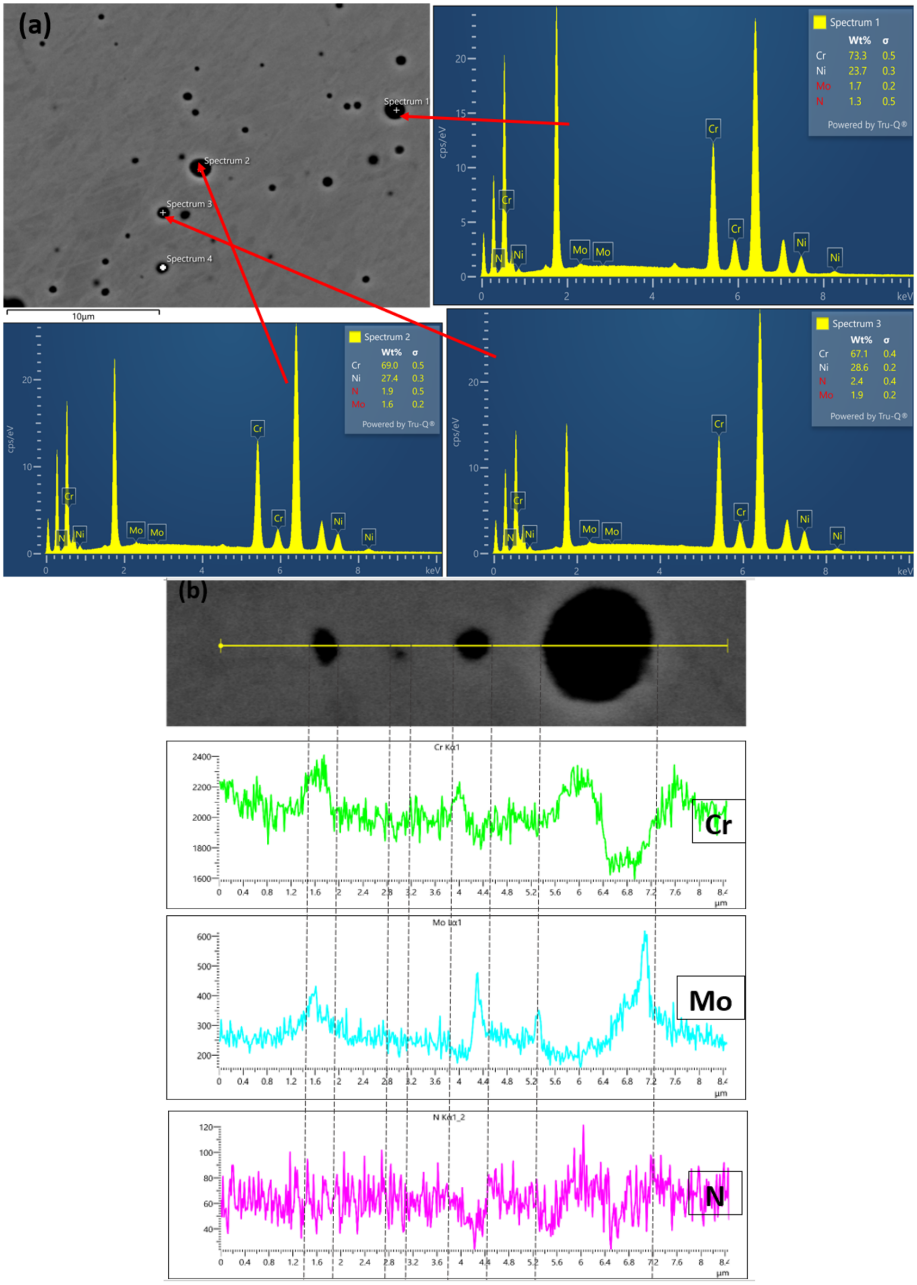
(**a**) SEM–EDS point analysis (1, 2, and 3) of the welded joint with E2 (**b**) EDS line mapping of the welded joint with E1 across the weldments to detect the presence of precipitates.

Figure 6a–c and e–g show the metallographic structure of welded DSS joints with E1 electrodes at various weld geometries (Fig. 6d) indicating where the optical micrographs were taken at different magnifications. Figure 6a, b, f are the transition zones of the welded joints signifying the ferrite–austenite phase balance structure. Figure 7a–c and e–g also show the OM of welded DSS joints with E2 electrodes at various weld geometries (Fig. 7d) signifying the point of OM analysis at different magnifications. Figure 7a, b, f are the transition zones of the welded joints signifying the ferrite–austenite phase balance. The OM at the weld zone (WZ) is illustrated in Figs. 6g and 7g respectively for the cases of welded joints with E1 and E2 electrodes. The OM at the BM is shown in Figs. 6c, e and 7c, e respectively for the cases of welded joints with E1 and E2 electrodes. The lighter zone refers to the austenite phase, and the dark-black zone typifies the ferrite phase. The phase balance of heat affected zone (HAZ) near the fusion line, which indicates the formation of Cr_2_N precipitates as can be seen in the SEM-BSE micrographs in Fig. 8a, b, confirmed by Fig. 9a, b. The emergence of Cr_2_N as observed in the ferrite phase of the samples in Fig. 8a, b and confirmed by SEM–EDS point analysis as well as EDS line mapping of the weldments (Fig. 9a–b), was occasioned by higher temperature during the weld thermal cycle which accelerated the chromium and nitrogen bonding because the diffusion coefficient of nitrogen is enhanced by high temperature in the weld. These findings corroborated with the studies of Ramirez et al.^68^ and Hereñú et al.^69^ that showed that Cr_2_N usually precipitates in ferrite grains, grains boundaries, and α/$\gamma$ interface regardless of the nitrogen content, as also opined by other researchers^70,71^.

**Figure 10:**
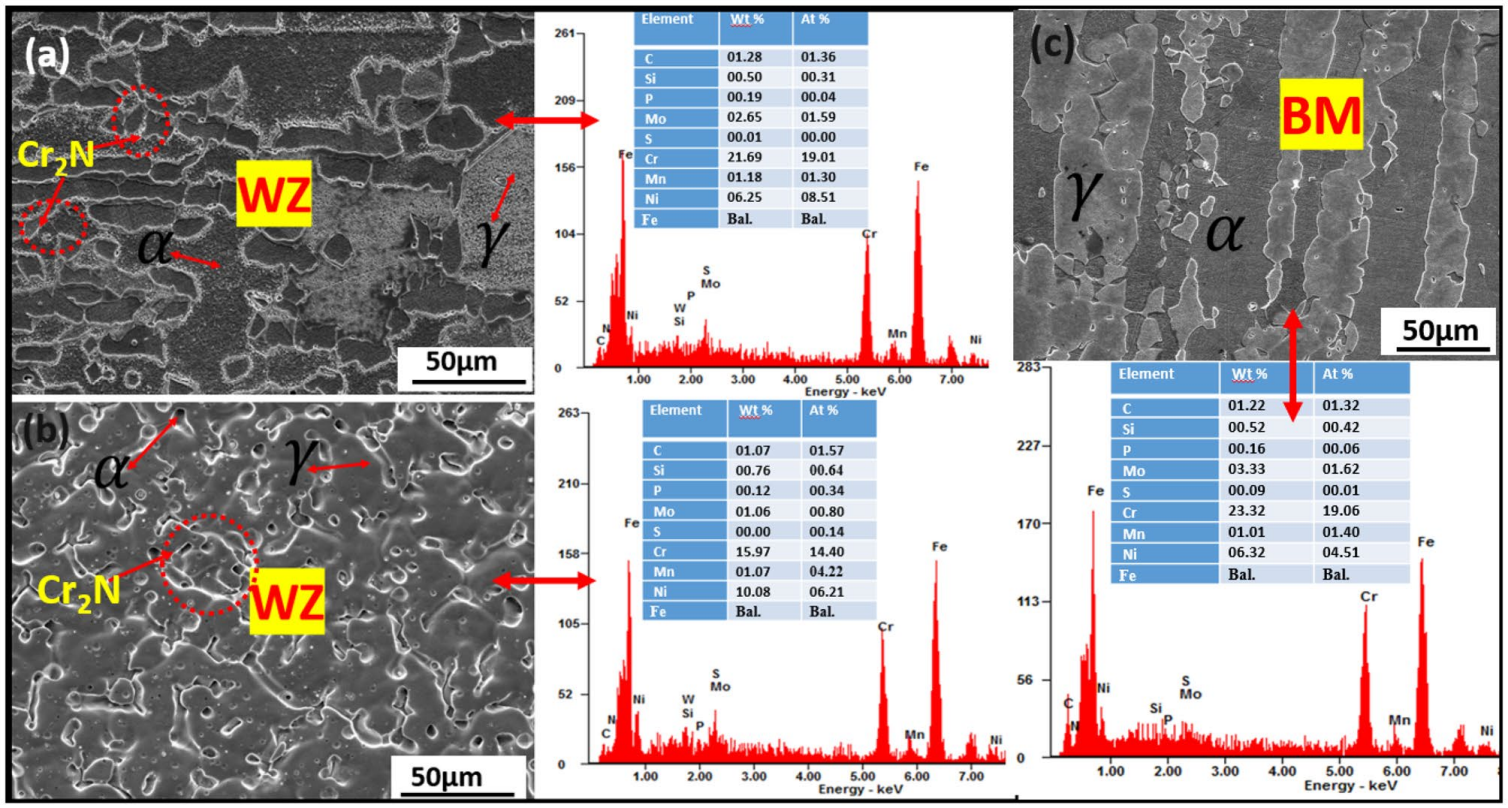
SEM micrographs of welded joint with (**a**) E1 electrode at weld zone with EDS spectra and (**b**) E2 electrode at weld zone with EDS spectra and (**c**) BM with EDS spectra.

The surface morphologies of representative samples are shown in Fig. 10a–c with their respective EDS. Figure 10a and b show the SEM micrographs of welded joint with E1 and E2 electrodes respectively at the weld zones with their EDS spectra, while Fig. 10c shows SEM micrograph of the BM with EDS spectra, comprising austenite ($\gamma$) and ferrite ($\alpha$) phases devoid of any precipitate. As evident in the EDS spectra of Fig. 10a is the percentage composition of chromium (21.69 wt.%) and molybdenum (2.65 wt.%) with 6.25 wt.% nickel that relatively gives a sense of ferrite–austenite phase balance in the corresponding microstructure as compared to the high reduction of the chromium (15.97 wt.%) and molybdenum contents (1.06 wt.%) as against the high nickel content (10.08 wt.%) in the microstructure of welded joints with E2 electrodes illustrated in Fig. 10b with the EDS spectra. The acicular shapes of the austenite structure with finer grains seen in the WZ shown in Fig. 10b, confirmed the eventual depletion of the ferritizing elements (Cr and Mo) at the weldment and precipitates of chromium nitride (Cr_2_N) along the interface of ferrite–austenite phases. This claim is corroborated by the distribution of precipitate particles along the boundaries of austenite ($\gamma$) and ferrite ($\alpha$) phases of welded DSS joints^72–74^. This also contributed to its poor corrosion performance since Cr is noted as the primary element used to form the passive film, that improves the localized corrosion resistance of the steel^59,75^ as also verified in Fig. 10b. It can be observed that the BM of the SEM micrograph of Fig. 10c shows strong grain refinements characteristics as its EDS spectra results depict good chemistry of Cr (23.32 wt.%), Mo (3.33 wt.%) and Ni (6.32 wt.%) as important alloying elements that validate the microstructures of the ferrite–austenite phase balance of DSS structure^76^. The results of the compositional EDS spectra analysis of welded joints with E1 electrode justify its applications in structural engineering as well as in mildly corrosive environments since both the austenite formers and ferrite stabilizer in the microstructures conform to standard welded joints of DSS AISI 2205^41,72,77^.

In actuality, it has been noted that DSSs welds solidify in fully ferritic mode (F mode) and austenite nucleates below the ferritic solvus temperature, which primarily depends on the chromium and nickel equivalent (Cr_eq_/Ni_eq_) ratio (> 1.95 is F mode) of the steel and has been noted by some researchers^78,79^ due to strong diffusivity of Cr and Mo as ferrite former elements in the ferrite phase^80^. It was evident that DSS 2205 BM possesses significant contents of Cr and Mo (exhibit higher Cr_eq_) yet it has less Ni content than welded joints with E1, E2, and C electrodes, which foster a higher Cr_eq_/Ni_eq_ ratio. This was also apparent in the current investigation where the determined Cr_eq_/Ni_eq_ ratio for DSS 2205 BM as indicated in Table 4 is greater than 1.95. It can be observed that welded joints with E1, E2, and C electrodes solidify as austenite-ferrite mode (A-F mode), austenite mode (A mode), and ferrite–austenite mode (F–A mode) respectively due to higher content of Ni and fewer contents of Cr and Mo in the weldment, signifying less ratio of Cr_eq_/Ni_eq_ than the BM as indicated in Table 4. The primary ferrite exhibited a vermicular-ferrite morphology in welded joint with E2 electrode and the determined Cr_eq_/Ni_eq_ ratio was 1.20 as mentioned in Table 4.

### Corrosion characterization

#### Potentiodynamic polarization analysis

**Figure 11:**
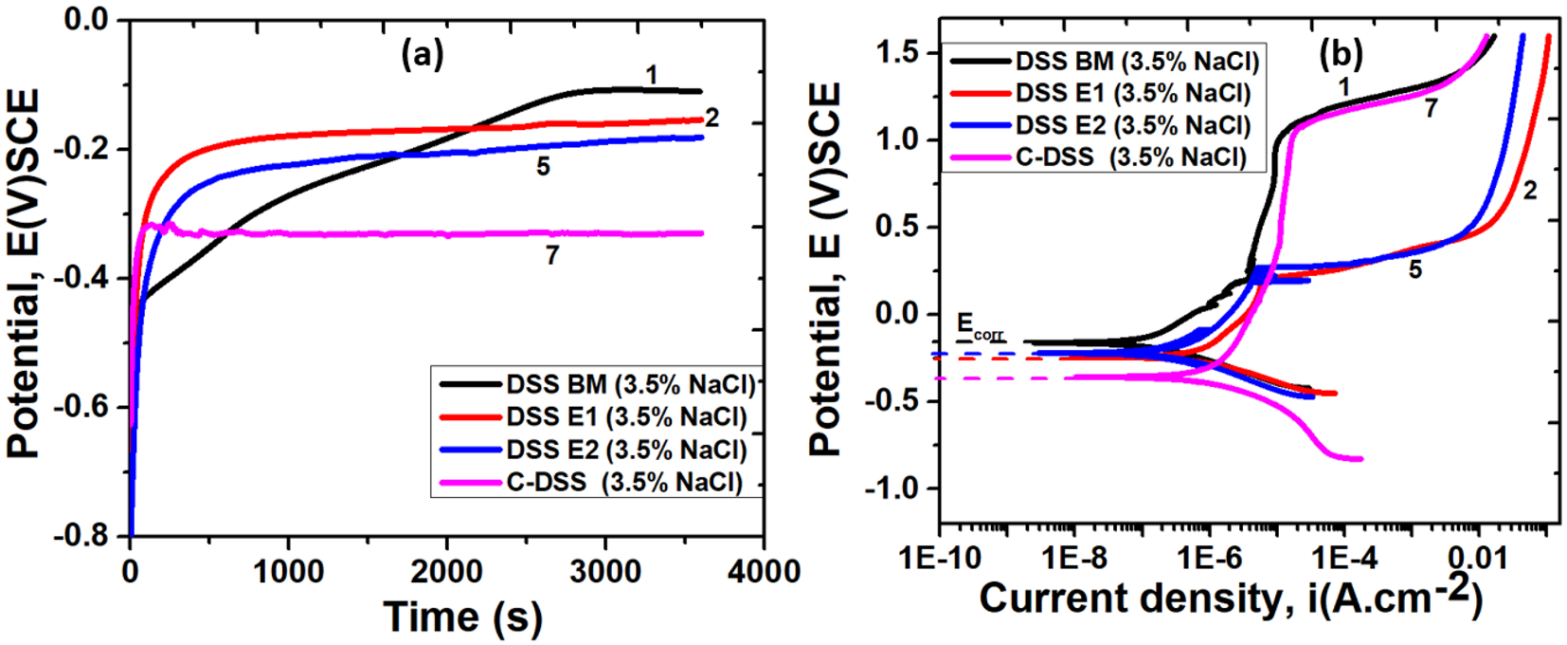
Electrochemical analysis of research samples: (**a**) Evolution of OCP vs. elapsed time and (**b**) Potentiodynamic polarization of samples in a solution of 3.5% NaCl.

Figure 11a shows the evolutions of the open circuit potential (OCP) scan with respect to time for AISI DSS 2205 steel structure in a 3.5% NaCl solution. It is evident that the OCP curve shifts toward more positive potentials, indicating the emergence of a passive film on the metal sample surface, and the decrease in potential indicates general corrosion, whereas potentials that remain nearly constant over time indicate that the passive film formed over the sample’s surface is steady and adhesive^77^. The curve depicts all the samples’ substrates for experimentation are in stable conditions in the electrolyte containing 3.5% NaCl solution except sample No. 7 (welded joint with C electrodes) which portrays slight instability. This instability could be likened to the presence of chloride ion (Cl^**-**^) in the solution that might have substantially accelerated the corrosion reaction and thereby increase the magnitude of corrosion. The observation in the OCP scan without applying potential shows that, Cl^-^ in the reaction may impact the resistance-ability and thermodynamic stability of the samples in the corrosive environment. This claim is corroborated by Ma et al.^81^, and Loto et al.^5^ on the effect of Cl^-^ in accelerating the passive film break-down of the substrate, thereby facilitating further deterioration.

**Figure 12:**
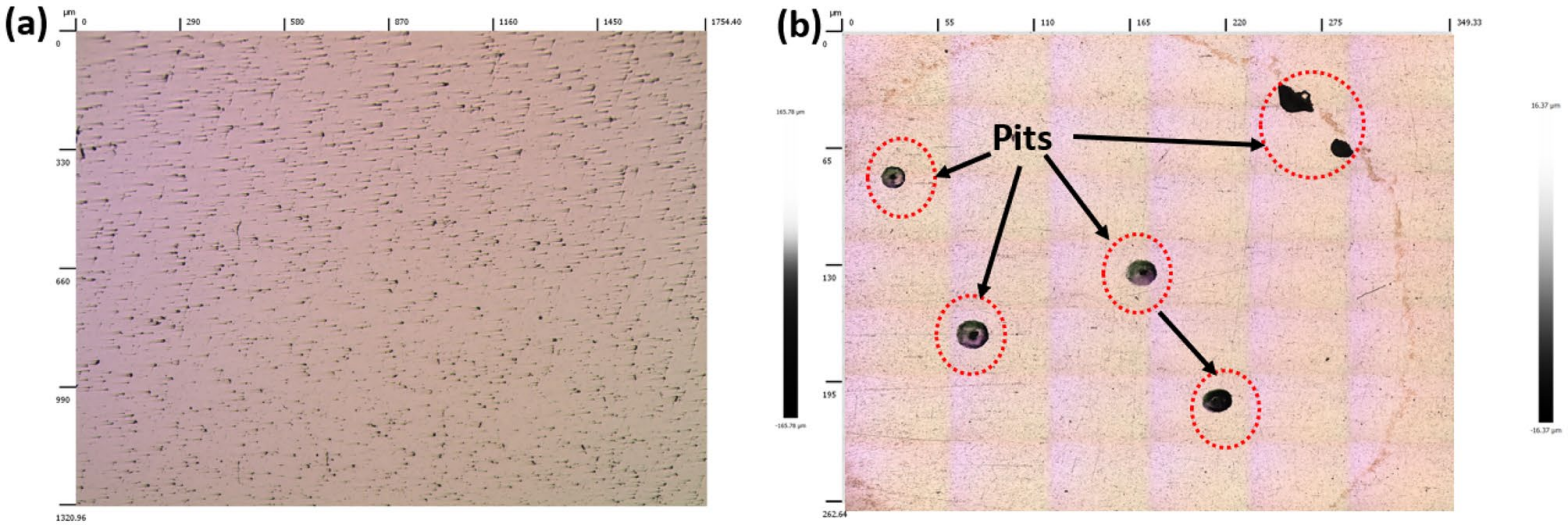
3D optical micrograph of AISI 2205 welded at welding current of (**a**) 90A and (**b**) 110A.

Figure 11b shows the comparative analysis of the potentiodynamic polarization curves (PPC) of welded joints with E1, E2, and C electrodes, exposed to a 3.5% NaCl solution. The PPC for BM and welded samples in 3.5% NaCl solution reveal a passive behaviour. Table 5 displays the parameters of electrochemical analysis of the samples obtained from PPC curves, such as *E*_*corr*_ (corrosion potential) and *E*_*pit*_ (pitting potential) with the associated deviations. Samples No. 1 and 7 (BM and welded joint with C electrode) demonstrated high pitting potentials in the solution of sodium chloride environments as compared to other samples No. 2 and 5 welded with E1 and E2 electrodes (Fig. 11b). The high passivation characteristics of the former than the latter is due to the microstructural compositional balance of the steel (austenite and ferrite phases) and the concentrations of the alloying elements. This assertion is supported by Rezendea et al.^82^ on the passive behaviour experienced by DSS in the corrosive environment due to the ferrite and austenite phases’ content of the microstructure. The low performance of welded samples with E1 and E2 electrodes may be due to the depletion of critical alloying elements like Cr and Mo at the weld zone (WZ) as the ferrite phase is stabilized by them (Cr and Mo) whereas they act as passivating alloys in the austenite phase of the steel. The effect of these elements in relation to the resistance to pit corrosion is greater in the austenite phase than in the ferrite phase. For this reason, the ferrite phase undergoes passivation more quickly than the austenite phase, associated with the first passive region of the polarization curve. These elements have a significant influence on the pitting resistance of DSS due to their higher pit corrosion resistance in the austenite phase as compared to the ferrite phase. Consequently, the quick passivation behaviour is experienced at the ferrite phase more than in the austenite counterpart^81^. Though the Cl^-^ in the solution has a strong detrimental effect on the passivating-ability of the steel film^83^. Therefore, the stability of the sample’s passive film would be reduced substantially^84^. It is also evident from Table 6 that the corrosion potentials (*E*_*corr*_) of welded joints with E1 electrodes seem to possess slight resistance to corrosion in the solution as compared to welded joints with E2 electrodes. This is further corroborated by the low hardness values noticed in the weldments with E1 and E2 electrodes from Fig. 4a, b, caused by their low ferrite fractions in the steel structure (Table 5) and low Cr and Mo composition (Table 4). It can be concluded that the resistance of the steel to corrosion in simulated sea environments increases with a decrease in welding current and decreases with low Cr and Mo compositions as well as low ferrite fractions. This assertion corresponds with the works of Salim et al.^85^ on the effects of welding parameters such as welding current on the corrosion integrity of welded steel. Because chloride penetrates steel by a variety of methods, including capillary absorption and diffusion, pits (pitting corrosion) with uneven shapes and varying depths form. The mechanism differed significantly in higher pH solutions where the (OH^-^) group in the environment is simply attracted to the steel surface, making the passive film stable and providing further protection to the steel surface^25,86^. The optimal corrosion resistance of samples No. 1 and 7 is largely due to the presence of large levels of delta ferrite (Table 5) and a substantial amount of Cr and Mo in the steel structure (Table 4), as the level of pitting is mostly found at the austenite phase structure of DSS weldments. Therefore, the chemistry of the alloy plays a crucial part in the corrosion properties of the weldment^87,88^. It was further observed that welded samples with E1 and C electrodes for this study, demonstrated low *E*_*corr*_ values from the PPC curve than welded joints with E2 electrodes from the OCP curve (Table 5). Consequently, the region of the anodic start at lower potentials. This variation can be ascribed primarily to the partial stabilization of the passive layer formed on the surface of the samples, as well as the cathodic polarization experienced prior to reaching total OCP stabilization^89^. As presented in Fig. 12a and b are the 3D optical profilometer images of the experimented corrosion samples under different welding conditions. It is obvious that the pitting size of the samples increases with a lower pitting potential which resulted from a high welding current of 110 A (Fig. 12b) in comparison with the pits size formations obtained from welded joints with a lower welding current of 90 A (Fig. 12a). This corroborated the assertion of Mohammed^90^ on the formation of slip band on the sample surface that disrupts the passive film on the surface, exposing the substrate to the working environment of 3.5% NaCl solution, thereby making the chloride begins attacking, leading to materials’ dissolution.

**Table 6:** Electrochemical parameters of DSS AISI 2205 in a 3.5% NaCl environment at a pH near 7.5.

Sample code	Sample no.	*E*_ocp_ (V)	*E*_corr_ (V)	*E*_pit_ (V)
DSS BM	1	− 0.2020	− 0.1638	1.0814 ± 6.97 × 10^–3^
DSS E1 @90A	2	− 0.3100	− 0.3390	0.3709 ± 7.97 × 10^–3^
DSS E1 @110A	3	− 0.1868	− 0.2338	0.2093 ± 5.89 × 10^–4^
DSS E2 @90A	4	− 0.2448	− 0.2415	0.3493 ± 3.48 × 10^–3^
DSS E2 @110A	5	− 0.2367	− 0.2320	0.2730 ± 2.55 × 10^–3^
C-DSS @90A	6	− 0.2800	− 0.3330	1.0670 ± 1.15 × 10^–2^
C-DSS @110A	7	− 0.3280	− 0.3530	1.0950 ± 1.96 × 10^–2^

The SEM–EDS analysis from Table 4 indicates that every austenite phase has a higher PREN value than the ferrite in all weldments and the BM. The breakdown of the passive layer of the material is accelerated by pitting nucleation at the ferrite/austenite interfaces due to the heterogeneity and elemental segregation that occurs in these areas^91^. As opposed to the austenite phase, where pitting resistance equivalent (PRE) values are higher, ferrite phase pitting nucleation is caused by lower PRE values (Table 4). The austenitic phase seems to possess significant austenite stabilizer (N solubility), allowing for substantial concentrations of this element and, consequently, higher pitting corrosion resistance^92^.

#### Critical pitting temperature

**Figure 13:**
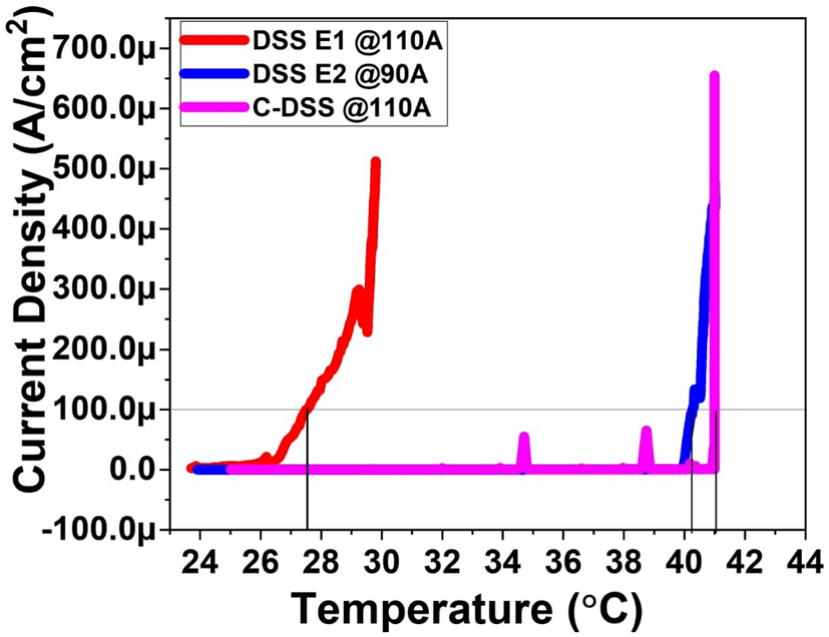
Critical pitting temperature of the welds with E1, E2, and C electrodes.

Figure 13 shows the critical pitting temperature curves for the welds E1, E2 and C. Considering the increase in current density due to pit formation to 100 µA/cm^2^ during the test as stated in the ASTM standard, it is evident that the weld with E1@110A showed lowest critical pitting temperature of 27.5 °C, followed by weld with E2@90A showed CPT of 40 °C and highest CPT of 41 °C in the case of C@110A. The observed results are well corroborated with observed polarization test results.

## Conclusions

The mechanical properties and corrosion behaviour of duplex stainless-steel weldment using novel electrodes E1 and E2 have been studied. Basic electrodes (E1) and acidic electrodes (E2) for the SMAW process were successfully coated from the formulated fluxes with a general coating factor of 1.7 mm and basicity indexes of 2.40 and 0.40 respectively. The thermal stability of the formulated flux using TGA in an inert environment was evaluated. The presence of high TiO_2_ (%) in the flux matrix enhances slag detachability in the weldment with the electrode coated with acidic flux (E2) than with the electrode coated with basic flux (E1). Though the two coated electrodes (E1 and E2) possess good arc striking-ability. Welding conditions especially heat input, welding current and speed play a crucial role in obtaining a balance of the austenite-ferrite phase of welded joints of DSS 2205 as well as excellent mechanical properties of the weldment. The excellent tensile properties shown by the joints welded with electrode E1 (0.2% offset YS = 497 MPa and UTS = 732 MPa) confirmed that electrodes coated with basic flux with high basicity index demonstrate better mechanical properties as compared to those coated with acidic flux with a low basicity index. It was obvious that the ferrite–austenite phase balance was lacking in the welded joints of the newly coated electrodes (E1 and E2) as revealed by OES and SEM–EDS analysis of the weldments and confirmed by volume fractions quantitative metallography in the weldment as well as their SEM examinations of the microstructures. This is largely due to the depletion of alloying elements like Cr and Mo and possible probable precipitation of Cr_2_N during welding as confirmed by the EDS line scan. The low hardness values observed in the weldments with E1 and E2 electrodes, caused by their low ferrite fractions and the alloying elements in the steel structure, are further validations. The evidential corrosion potentials (*E*_*corr*_) of welded joints with E1 electrodes seem to possess slight resistance to corrosion in the solution as compared to welded joints with E2 electrodes. This validates the performance of the welded joints made by the newly developed electrode without alloying components from the flux mixture tested in a 3.5% of NaCl environment. It can be concluded that the resistance to corrosion in simulated sea environments increases with a decrease in welding current. Hence, the carbide and nitride evolutions, and the consequent reduction of corrosion resistance-ability of the weldments using E1 and E2 electrodes are attributed to increased welding current resulting in an unbalanced phase equilibrium of the dual-purpose steel weldments.

## Data Availability

On request, the implemented data for this research will be made available by the corresponding author.
